# Strain-Dependent Prion Infection in Mice Expressing Prion Protein with Deletion of Central Residues 91–106

**DOI:** 10.3390/ijms21197260

**Published:** 2020-10-01

**Authors:** Keiji Uchiyama, Hironori Miyata, Yoshitaka Yamaguchi, Morikazu Imamura, Mariya Okazaki, Agriani Dini Pasiana, Junji Chida, Hideyuki Hara, Ryuichiro Atarashi, Hitomi Watanabe, Gen Kondoh, Suehiro Sakaguchi

**Affiliations:** 1 Division of Molecular Neurobiology, The Institute for Enzyme Research (KOSOKEN), Tokushima University, 3-18-15 Kuramoto, Tokushima 770-8503, Japan; ku200@tokushima-u.ac.jp (K.U.); y-yama@mimasaka.ac.jp (Y.Y.); c201801078@tokushima-u.ac.jp (M.O.); agrianipasiana@yahoo.com (A.D.P.); jchida@tokushima-u.ac.jp (J.C.); hara@tokushima-u.ac.jp (H.H.); 2 Animal Research Center, School of Medicine, University of Occupational and Environmental Health, Yahatanishi, Kitakyushu 807-8555, Japan; h-miyata@med.uoeh-u.ac.jp; 3 Division of Microbiology, Department of Infectious Diseases, Faculty of Medicine, University of Miyazaki, 5200 Kihara, Kiyotake, Miyazaki 889-1692, Japan; morikazu_imamura@med.miyazaki-u.ac.jp (M.I.); ryuichiro_atarashi@med.miyazaki-u.ac.jp (R.A.); 4 Student Laboratory, Tokushima University, Faculty of Medicine, 3-18-15 Kuramoto, Tokushima 770-8503, Japan; 5 Laboratory of Integrative Biological Science, Institute for Frontier Life and Medical Sciences, Kyoto University, Yoshida-Konoe-cho, Sakyo-ku, Kyoto 606-8501, Japan; watanabe@frontier.kyoto-u.ac.jp (H.W.); kondohg@frontier.kyoto-u.ac.jp (G.K.)

**Keywords:** prions, prion protein, protein misfolding, neurodegeneration, transgenic mice

## Abstract

Conformational conversion of the cellular prion protein, PrP^C^, into the abnormally folded isoform, PrP^Sc^, is a key pathogenic event in prion diseases. However, the exact conversion mechanism remains largely unknown. Transgenic mice expressing PrP with a deletion of the central residues 91–106 were generated in the absence of endogenous PrP^C^, designated Tg(PrP∆91–106)/*Prnp^0/0^* mice and intracerebrally inoculated with various prions. Tg(PrP∆91–106)/*Prnp^0/0^* mice were resistant to RML, 22L and FK-1 prions, neither producing PrP^Sc^∆91–106 or prions in the brain nor developing disease after inoculation. However, they remained marginally susceptible to bovine spongiform encephalopathy (BSE) prions, developing disease after elongated incubation times and accumulating PrP^Sc^∆91–106 and prions in the brain after inoculation with BSE prions. Recombinant PrP∆91-104 converted into PrP^Sc^∆91–104 after incubation with BSE-PrP^Sc^-prions but not with RML- and 22L–PrP^Sc^-prions, in a protein misfolding cyclic amplification assay. However, digitonin and heparin stimulated the conversion of PrP∆91–104 into PrP^Sc^∆91–104 even after incubation with RML- and 22L-PrP^Sc^-prions. These results suggest that residues 91–106 or 91–104 of PrP^C^ are crucially involved in prion pathogenesis in a strain-dependent manner and may play a similar role to digitonin and heparin in the conversion of PrP^C^ into PrP^Sc^.

## 1. Introduction

The normal cellular isoform of prion protein, designated PrP^C^, is a glycoprotein tethered to the outer cell membrane via a glycosylphosphatidylinositol (GPI) anchor moiety and expressed most abundantly in the brain, particularly by neurons [1,2]. Its conformational conversion into the abnormally folded amyloidogenic isoform, PrP^Sc^, which is believed to be a major component of infectious prions, is a key pathogenic event in prion diseases, a group of neurodegenerative disorders, including Creutzfeldt-Jakob disease in humans and scrapie and bovine spongiform encephalopathy (BSE) in animals [1,2]. However, the exact conversion mechanism remains largely unknown.

Mice lacking PrP^C^ (*Prnp^0/0^*) have been shown to be resistant to prion infection, neither producing PrP^Sc^ or prions in the brain nor developing disease even after intracerebral inoculation with the prions [3,4,5,6]. Reverse genetic studies using reconstituted *Prnp^0/0^* mice with various transgenes encoding mutant PrP molecules have revealed that the N-terminal domain plays an important role in the conversion of PrP^C^ into PrP^Sc^ after prion infection. Indeed, *Prnp^0/0^* mice expressing PrP with a deletion of the N-terminal residues 23–31, designated Tg(PrP∆23–31)/*Prnp^0/0^* mice, were shown to be highly resistant to RML scrapie prions, developing disease only after longer incubation times and showing a slower accumulation of pathogenic PrP or PrP^Sc^∆23–31, in the brain after infection with RML scrapie prions [7]. In contrast, Tg(PrP∆32–80)/*Prnp^0/0^* mice, which express PrP with a deletion of residues 32–80, developed disease without an elongated incubation time and accumulated PrP^Sc^∆23–80 in the brain after infection with RML prions [8]. Moreover, *Prnp^0/0^* mice expressing PrP with a deletion of residues 51–90, which correspond to the so-called octapeptide repeat (OR) region, were also susceptible to RML and 22L scrapie prions, developing disease without an elongated incubation time and accumulating PrP^Sc^∆OR in the brain [9,10]. These results indicate that, in contrast to residues 23–31, residues from position 32 to 90 may be dispensable for the conversion of PrP^C^ into PrP^Sc^, as well as to support prion infection. However, Tg(PrP∆32–93)/*Prnp^0/0^* mice expressing PrP with a deletion extended to position 93 developed disease with longer incubation times with lower levels of prion infectivity and PrP^Sc^∆32–93 in the brain after infection with RML prions [11]. Furthermore, PrP with a deletion further extended to position 106 or PrP∆32–106, failed to convert into PrP^Sc^ or support prion pathogenesis in *Prnp^0/0^* mice after intracerebral inoculation with RML prions [12]. These results suggest that residues 91–106, which are completely deleted in PrP∆32–106 and partially in PrP∆32–93 but intact in PrP∆32–80 and PrP∆OR, could play a role in the conversion of PrP^C^ into PrP^Sc^ and prion pathogenesis after prion infection. However, this remains to be clarified.

In this study, to evaluate the role of residues 91–106 of PrP^C^ in prion pathogenesis, Tg(PrP∆91–106)/*Prnp^0/0^* mice expressing mouse PrP lacking residues 91–106 or PrP∆91–106, on the *Prnp^0/0^* background were generated and intracerebrally inoculated with mouse-adapted scrapie prions of RML and 22L, mouse-adapted human prions of FK-1 and mouse-adapted BSE prions. Tg(PrP∆91–106)/*Prnp^0/0^* mice were found to be resistant to RML, 22L and FK-1 prions, neither accumulating PrP^Sc^∆91–106 or infectious prions in the brain nor developing disease after inoculation. However, they were found to be marginally susceptible to BSE prions, succumbing to disease after long incubation times, as well as accumulating PrP^Sc^∆91–106 and propagating BSE prions in the brain after inoculation. Furthermore, using an in vitro protein misfolding cyclic amplification (PMCA) assay, recombinant PrP with a smaller deletion of residues 91–104 or PrP∆91–104, was converted into PrP^Sc^∆91–104 after incubation with BSE-PrP^Sc^-prions but not with RML- and 22L-PrP^Sc^-prions. Interestingly, digitonin and heparin stimulated the conversion of PrP∆91–104 into PrP^Sc^∆91–104 even after incubation with RML- and 22L-PrP^Sc^-prions. These results suggest that residues 91–106 or 91–104 of PrP^C^ are crucial for prion pathogenesis in a strain-dependent manner and may play a similar role to digitonin and heparin in the conversion of PrP^C^ into PrP^Sc^.

## 2. Results

### 2.1. Generation of Tg(PrP∆91–106)/Prnp^0/0^ Mice

**Figure 1:**
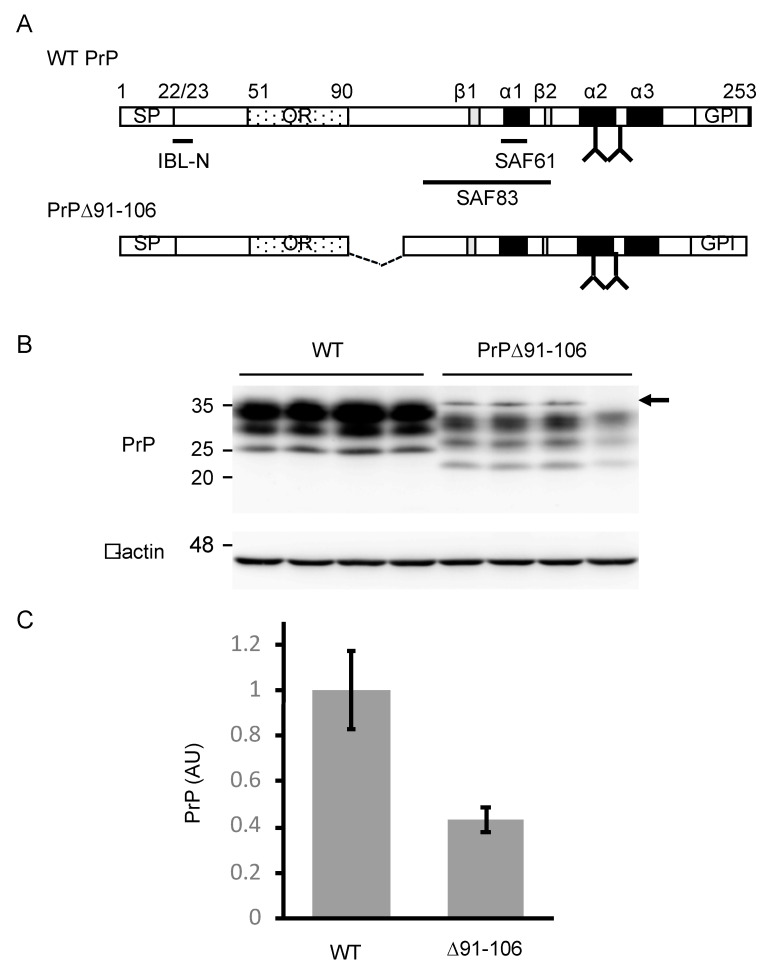
Generation of Tg(PrP∆91–106)/*Prnp^0/0^* mice. (**A**) Schematic diagrams of wild-type (WT) PrP and PrP∆91–106. The epitopes of IBL-N, SAF61 and SAF83 anti-PrP antibodies are shown. Arabic number, codon number; SP, signal peptide; OR, octapeptide repeat region; GPI, GPI anchor signal; α, α-helix; β, β-strand; Y, glycosylation site. (**B**) Western blotting (15% SDS-polyacrylamide gel (SDS-PAG)) of the brains (40 μg of total proteins) of WT (*n* = 4) and Tg(PrP∆91–106)/*Prnp^0/0^* (*n* = 4) mice with IBL-N anti-PrP antibodies. An arrow indicates non-specific signals. β-actin was used as an internal control (15 μg of total proteins, 15% SDS-PAG). (**C**) Densitometric analysis of WT PrP^C^ and PrP∆91–106 in the brains of WT and Tg(PrP∆91–106)/*Prnp^0/0^* mice in (**B**) after normalization against β-actin. AU, arbitrary units. Data are represented as the mean ± standard deviation (SD).

To investigate the role of residues 91–106 in the conversion of PrP^C^ into PrP^Sc^ and prion pathogenesis, we produced Tg(PrP∆91–106)/*Prnp^0/0^* mice. A cDNA encoding PrP∆91–106 (Figure 1A) was introduced into the Syrian hamster PrP cosmid vector, CosSHa.tet [13], which allows the mutant protein to be expressed under the control of the hamster PrP promoter. The transgene was then microinjected into the fertilized eggs of C57BL/6 wild-type (WT) mice, yielding a line of Tg(PrP∆91–106) mice. To eliminate the endogenous expression of WT PrP^C^ in the Tg mice, the Tg mice were successively intercrossed with *Prnp^0/0^* mice, resulting in Tg(PrP∆91–106)/*Prnp^0/0^* mice. Tg(PrP∆91–106)/*Prnp^0/0^* mice were born without obvious developmental abnormalities and grew normally, based on inspection more than two years, suggesting that PrP∆91–106 might not be toxic in mice. To investigate the expression levels of PrP∆91–106 in the brain of Tg(PrP∆91–106)/*Prnp^0/0^* mice, we carried out western blotting of the brain homogenates from C57BL/6 WT and Tg(PrP∆91–106)/*Prnp^0/0^* mice with IBL-N anti-PrP antibodies, which were raised against the N-terminal peptide of PrP. The antibodies showed PrP^C^ and PrP∆91–106 expressed in WT and Tg(PrP∆91–106)/*Prnp^0/0^* mice, respectively (Figure 1B and Supplementary Figure S1). PrP∆91–106 appeared to migrate faster than WT PrP^C^ in the WT mice, with three major bands similar to those of PrP^C^, each corresponding to di-glycosylated, mono-glycosylated and non-glycosylated forms (Figure 1B and Supplementary Figure S1). The di-glycosylated form was predominantly expressed, followed by the mono-glycosylated form and the non-glycosylated form. Densitometric analysis indicated that the levels of PrP∆91–106 expressed in the brains of Tg(PrP∆91–106)/*Prnp^0/0^* mice were about 0.4-fold as high as in PrP^C^ in WT mice (Figure 1C).

### 2.2. PrP∆91–106 Does Not Alter Its Localization at Raft Micromembrane Domains

**Figure 2:**
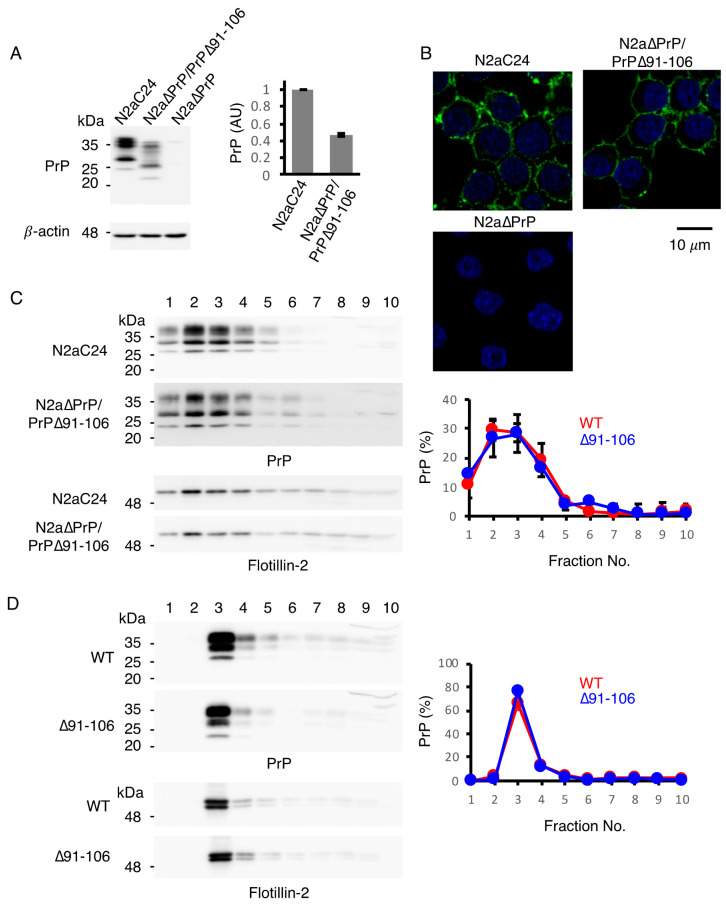
Subcellular localization of PrP∆91–106 at raft micromembrane domains. (**A**) Left: Western blotting (15% SDS-PAG) of N2aC24, N2a∆PrP/PrP∆91–106 and N2a∆PrP cells for WT PrP^C^ and PrP∆91–106 with IBL-N anti-PrP antibodies (40 μg of total proteins). β-actin was used as an internal control (15 μg of total proteins, 15% SDS-PAG). Right: Densitometric analysis of WT PrP^C^ and PrP∆91–106 in N2aC24 and N2a∆PrP/PrP∆91–106 cells after normalization against β-actin. AU, arbitrary units. Data are presented as the mean ± SD of three independent experiments. (**B**) Immunofluorescence staining of WT PrP^C^ and PrP∆91–106 in permeabilized N2aC24, N2a∆PrP/PrP∆91–106 and N2a∆PrP cells with SAF83 anti-PrP antibody. WT PrP^C^ and PrP∆91–106, green; nucleus, blue (DAPI staining). (**C**) Left: Western blotting of N2aC24 and N2a∆PrP/PrP∆91–106 cells subjected to discontinuous sucrose gradient centrifugation for WT PrP^C^ and PrP∆91–106 with IBL-N anti-PrP antibodies (15% SDS-PAG) and a raft domain marker flotillin-2 (10% SDS-PAG). About 20% of each fraction were applied to western blotting. Right: Quantification of WT PrP^C^ and PrP∆91–106 in each fraction against the total WT PrP^C^ and PrP∆91–106. The signal density in each lane was evaluated against the total signal density of all lanes. Data are presented as the mean ± SD of three independent experiments. (**D**) Left: Western blotting of WT and Tg(PrP∆91–106)/*Prnp^0/0^* mouse brains subjected to discontinuous sucrose gradient centrifugation for WT PrP^C^ and PrP∆91–106 with IBL-N anti-PrP antibodies (15% SDS-PAG) and flotillin-2 (10% SDS-PAG). About 3% of each fraction were applied to western blotting. Right: Quantification of WT PrP^C^ and PrP∆91–106 in each fraction against the total WT PrP^C^ and PrP∆91–106. The signal density in each lane was evaluated against the total signal density of all lanes. Data are presented as the mean ± SD of 3 independent experiments.

To confirm whether PrP∆91–106 locates to the lipid raft micromembrane domains similarly to PrP^C^, we compared the subcellular localization of PrP∆91–106 with that of PrP^C^ in mouse neuroblastoma N2a cells using immunofluorescent staining. To this end, we established a cell line, termed N2a∆PrP/PrP∆91–106, which constitutively expressed PrP∆91–106 in the absence of endogenous WT PrP^C^, by introducing an expression vector encoding PrP∆91–106 into PrP^C^-knockout N2a cells, designated N2a∆PrP cells [14]. As a control, we used N2a cells expressing exogenous mouse PrP^C^, termed N2aC24 cells [15]. Similar to Tg(PrP∆91–106)/*Prnp^0/0^* mice, PrP∆91–106 was expressed at lower levels in N2a∆PrP/PrP∆91–106 cells compared to WT PrP^C^ in N2aC24 cells on western blotting with IBL-N anti-PrP antibodies (Figure 2A and Supplementary Figure S2A). Immunofluorescent staining with SAF83 anti-PrP antibody, which recognizes residues 125–163 of mouse PrP, showed that PrP^C^ was predominantly expressed on the surface of N2aC24 cells (Figure 2B). This is consistent with PrP^C^ being a GPI-anchored membrane glycoprotein. Similar cell surface staining was also observed for PrP∆91–106 in PrP∆PrP/PrP∆91–106 cells (Figure 2B). Next, a sucrose density gradient assay was performed for both cell lysates to elucidate the microdomain localization of PrP^C^ and PrP∆91–106 at the cell membrane. Western blotting with IBL-N anti-PrP antibodies showed that both molecules were predominantly detected at the raft domain fractions (93.4 ± 6.0% for PrP^C^, 83.2 ± 11.5% for PrP∆91–106), which were represented by the presence of a raft protein, flotillin-2 (Figure 2C and Supplementary Figure S2B). We also performed a sucrose density gradient assay for the brain homogenates from WT and Tg(PrP∆91–106)/*Prnp^0/0^* mice. PrP^C^ and PrP∆91–106 were also predominantly localized at the raft domains in the mouse brain (88.5 ± 2.0% for PrP^C^, 93.5 ± 1.3% for PrP∆91–106; Figure 2D and Supplementary Figure S2C). These results confirm that PrP∆91–106 and WT PrP^C^ were similarly localized at the raft microdomains on the cell surface, indicating that deletion of residues 91–106 does not affect the subcellular localization of PrP∆91–106 at the raft membrane domains on the cell surface.

### 2.3. Tg(PrP∆91–106)/Prnp^0/0^ Mice Are Resistant to RML, 22L and FK-1 Prions but Still Susceptible to BSE Prions

**Figure 3:**
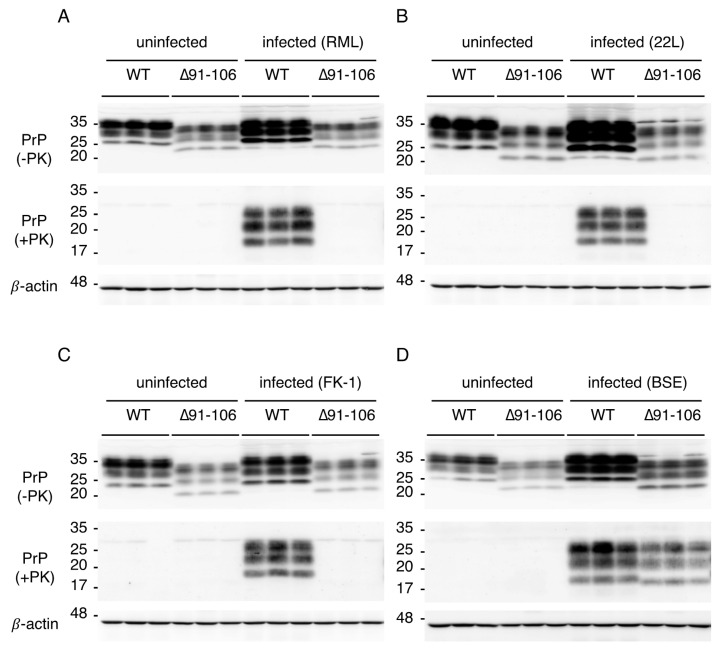
Accumulation of PrP^Sc^∆91–106 in the brains of Tg(PrP∆91–106)/*Prnp^0/0^* mice inoculated with BSE prions. (**A**) Western blotting (15% SDS-PAG) of the brains of uninfected WT mice (*n* = 3), uninfected Tg(PrP∆91–106)/*Prnp^0/0^* mice (*n* = 3), RML-infected, terminally ill WT mice (*n* = 3; 155, 163 and 178 dpi) and RML-inoculated, asymptomatic Tg(PrP∆91–106)/*Prnp^0/0^* mice (*n* = 3; 573 dpi) with SAF61 anti-PrP antibody after treatment with (+) and without (−) PK (40 μg of total proteins for PK−, 50 μg of total proteins for PK+). β-actin was used as an internal control (15 μg of total proteins, 15% SDS-PAG). (**B**) Western blotting (15% SDS-PAG) of the brains of uninfected WT mice (*n* = 3), uninfected Tg(PrP∆91–106)/*Prnp^0/0^* mice (*n* = 3), 22L-infected, terminally ill WT mice (*n* = 3; 156, 156 and 158 dpi) and 22L-inoculated, asymptomatic Tg(PrP∆91–106)/*Prnp^0/0^* mice (*n* = 3; 603 dpi) with SAF61 anti-PrP antibody after treatment with (+) and without (‒) PK (40 μg of total proteins for PK−, 50 μg of total proteins for PK+). β-actin was used as an internal control (15 μg of total proteins, 15% SDS-PAG). (**C**) Western blotting (15% SDS-PAG) of the brains of uninfected WT mice (*n* = 3), uninfected Tg(PrP∆91–106)/*Prnp^0/0^* mice (*n* = 3), FK-1-infected, terminally ill WT mice (*n* = 3; 155, 157 and 203 dpi) and FK-1-inoculated, asymptomatic Tg(PrP∆91–106)/*Prnp^0/0^* mice (*n* = 3; 603 dpi) with SAF61 anti-PrP antibody after treatment with (+) and without (‒) PK (40 μg of total proteins for PK−, 50 μg of total proteins for PK+). β-actin was used as an internal control (15 μg of total proteins, 15% SDS-PAG). (**D**) Western blotting (15% SDS-PAG) of the brains of uninfected WT mice (*n* = 3), uninfected Tg(PrP∆91–106)/*Prnp^0/0^* mice (*n* = 3), BSE-infected, terminally ill WT (*n* = 3; 167, 172 and 172 dpi) and Tg(PrP∆91–106)/*Prnp^0/0^* mice (*n* = 3; 455, 455 and 469 dpi) with SAF61 anti-PrP antibody after treatment with (+) and without (‒) PK (40 μg of total proteins for PK−, 50 μg of total proteins for PK+). β-actin is an internal control (15 μg of total proteins, 15% SDS-PAG).

To investigate the role of residues 91–106 of PrP^C^ in prion infection, we intracerebrally inoculated Tg(PrP∆91–106)/*Prnp^0/0^* and C57BL/6 WT mice with RML, 22L, FK-1 and BSE prions. WT mice developed disease at 171 ± 4, 143 ± 1, 187 ± 3 and 174 ± 4 days post-inoculation (dpi) with RML, 22L, FK-1 and BSE prions, respectively (Table 1). However, none of the Tg(PrP∆91–106)/*Prnp^0/0^* mice inoculated with RML, 22L and FK-1 prions succumbed to disease (Table 1). They were also highly resistant, although marginally susceptible, to BSE prions, developing disease only after very long incubation times (538 ± 24 dpi) (Table 1). We then inspected the brains of WT and Tg(PrP∆91–106)/*Prnp^0/0^* mice inoculated with RML, 22L, FK-1 and BSE prions for PrP^Sc^ and PrP^Sc^∆91–106 by western blotting using SAF61 anti-PrP antibody after treatment with proteinase K (PK). WT mice accumulated PrP^Sc^ in the brain at the terminal stages of infection with RML, 22L, FK-1 and BSE prions (155, 163 and 178 dpi with RML prions; 156, 156 and 158 dpi with 22L prions; 155, 157 and 203 dpi with FK-1 prions, 167, 172 and 172 dpi with BSE prions; Figure 3A–D and Supplementary Figure S3A–D). No PrP^Sc^∆91–106 was detectable in the brains of Tg(PrP∆91–106)/*Prnp^0/0^* mice, which were sacrificed at 573 dpi with RML prions, 603 dpi with 22L prions and 603 dpi with FK-1 prions (Figure 3A–C and Supplementary Figure S3A–C). These time points were later than BSE-inoculated Tg(PrP∆91–106)/*Prnp^0/0^* mice developed disease. In contrast, PrP^Sc^∆91–106 accumulated in the brains of BSE-infected, terminally ill Tg(PrP∆91–106)/*Prnp^0/0^* mice (455, 455 and 469 dpi; Figure 3D and Supplementary Figure S3D). These results indicate that Tg(PrP∆91–106)/*Prnp^0/0^* mice are still susceptible to BSE prions but not to RML, 22L and FK-1 prions.

**Figure 4:**
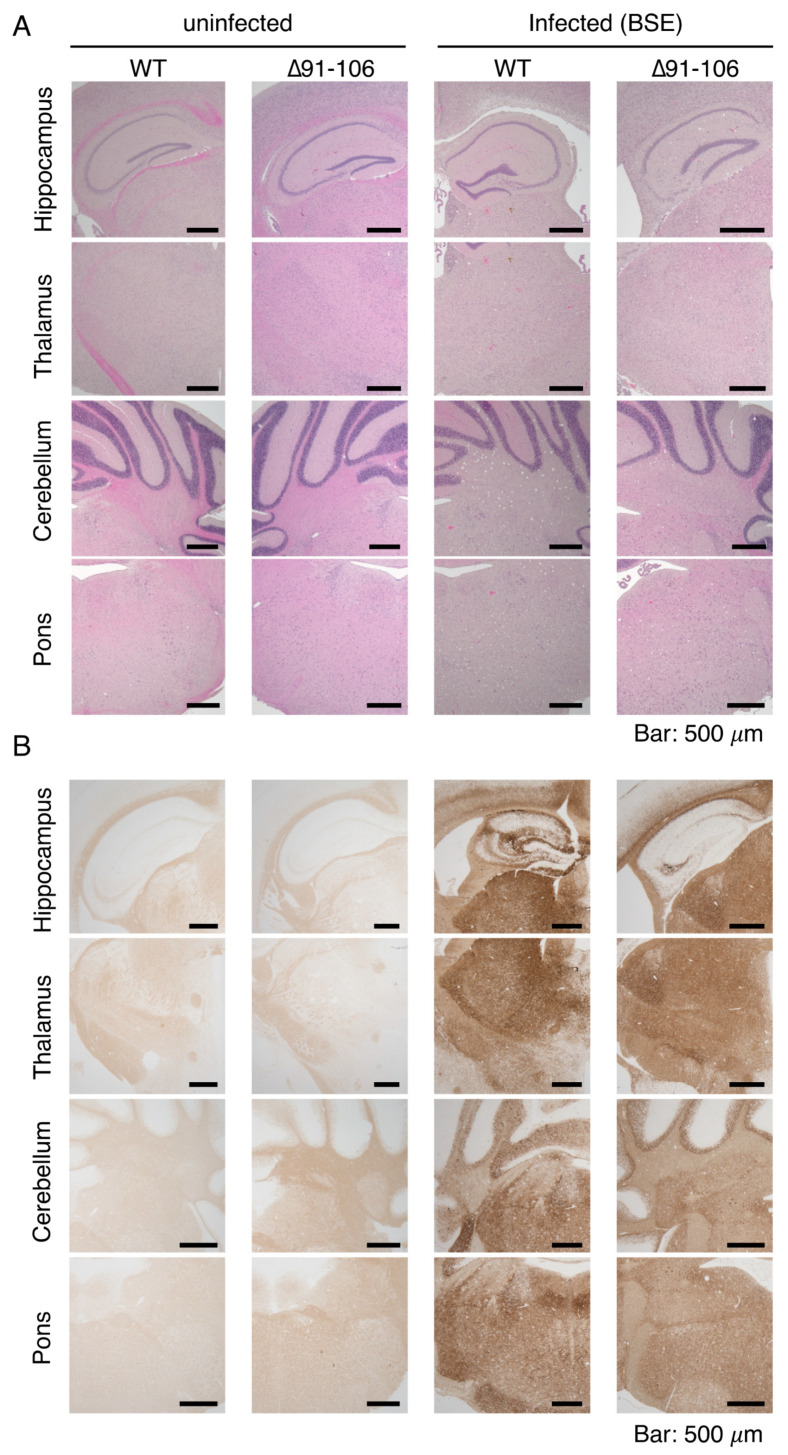

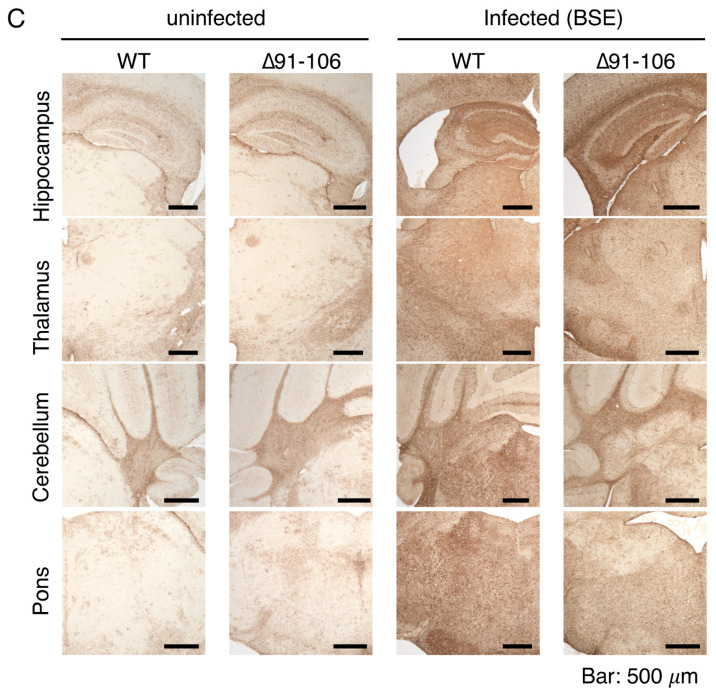
Pathological examinations of the brains of BSE-infected, terminally ill Tg(PrP∆91–106)/*Prnp^0/0^* mice. (**A**) Hematoxylin-eosin (HE) staining of the brains of uninfected WT and Tg(PrP∆91–106)/*Prnp^0/0^* mice and BSE-infected, terminally ill WT and Tg(PrP∆91–106)/*Prnp^0/0^* mice. (**B**) Immunohistochemistry for WT PrP^Sc^ and PrP^Sc^∆91–106 with SAF83 anti-PrP antibody in the brains of uninfected WT and Tg(PrP∆91–106)/*Prnp^0/0^* mice and BSE-infected, terminally ill WT and Tg(PrP∆91–106)/*Prnp^0/0^* mice. (C) Immunohistochemistry for glial fibrillar acidic protein (GFAP) with anti-GFAP antibody in the brains of uninfected WT and Tg(PrP∆91–106)/*Prnp^0/0^* mice and BSE-infected, terminally ill WT and Tg(PrP∆91–106)/*Prnp^0/0^* mice.

The brains of BSE-infected, terminally ill Tg(PrP∆91–106)/*Prnp^0/0^* mice were then pathologically investigated. Hematoxylin-eosin (HE) staining showed disease-specific vacuolation throughout the brain slices of BSE-infected, terminally ill Tg(PrP∆91–106)/*Prnp^0/0^* mice, particularly abundant in the cerebellum and pons, intermediately in the thalamus and mildly in the hippocampus, similar to BSE-infected, terminally WT mice (Figure 4A). No vacuolation was observed in the brains of uninfected Tg(PrP∆91–106)/*Prnp^0/0^* mice aged 424–636 days old (*n* = 5), supporting that PrP∆91–106 might not be toxic. PrP^Sc^ and PrP^Sc^∆91–106 were found to be accumulated throughout the brain slices of BSE-infected, terminally ill WT and Tg(PrP∆91–106)/*Prnp^0/0^* mice using SAF83 anti-PrP antibody, respectively (Figure 4B). Staining for glial fibrillar acidic protein (GFAP) also showed similarly marked astrocytosis in the brains between BSE-infected, terminally ill WT and Tg(PrP∆91–106)/*Prnp^0/0^* mice (Figure 4C). These results indicate that disease-specific brain pathologies may not be altered in BSE-infected Tg(PrP∆91–106)/*Prnp^0/0^* mice, suggesting that the tropism of BSE prions is not changed in the brain of Tg(PrP∆91–106)/*Prnp^0/0^* mice. Neither vacuolation nor PrP^Sc^ accumulation was detected in the brains of Tg(PrP∆91–106)/*Prnp^0/0^* mice sacrificed at 573 dpi with RML prions, 603 dpi with 22L prions and 603 dpi FK-1 prions (Supplementary Figure S4A–C).

### 2.4. Tg(PrP∆91–106)/Prnp^0/0^ Mice Propagate BSE Prions but Not RML, 22L, FK-1 Prions

To rule out the possibility that Tg(PrP∆91–106)/*Prnp^0/0^* mice may be susceptible to RML, 22L and FK-1 prions, propagating them in the brain after inoculation at levels not high enough for the detection of PrP^Sc^∆91–106 by western blotting, we intracerebrally inoculated the brain homogenates from Tg(PrP∆91–106)/*Prnp^0/0^* mice sacrificed at 922 dpi with RML prions, 884 dpi with 22L prions or 604 dpi with FK-1 prions into Tg(PrP∆91–106)/*Prnp^0/0^* and C57BL/6 WT mice. None of Tg(PrP∆91–106)/*Prnp^0/0^* or WT mice succumbed to disease up to 730 or 732 dpi with RML-, 22L- and FK-1-inoculated Tg(PrP∆91–106)/*Prnp^0/0^* brain homogenates (Table 2). We also intracerebrally inoculated Tg(PrP∆91–106)/*Prnp^0/0^* and WT mice with brain homogenates from BSE-infected, terminally ill Tg(PrP∆91–106)/*Prnp^0/0^* mice. Tg(PrP∆91–106)/*Prnp^0/0^* and WT mice developed disease at 343 ± 28 and 326 ± 29 dpi with BSE-infected Tg(PrP∆91–106)/*Prnp^0/0^* brain homogenates, respectively (Table 2). These results further confirm that the Tg(PrP∆91–106)/*Prnp^0/0^* mice are resistant to RML, 22L and FK-1 prions but not to BSE prions.

### 2.5. Digitonin and Heparin Stimulate the Conversion of PrP∆91–104 into PrP^Sc^∆91–104 Even after Incubation with RML- and 22L-PrP^Sc^-Prions In Vitro

**Figure 5:**
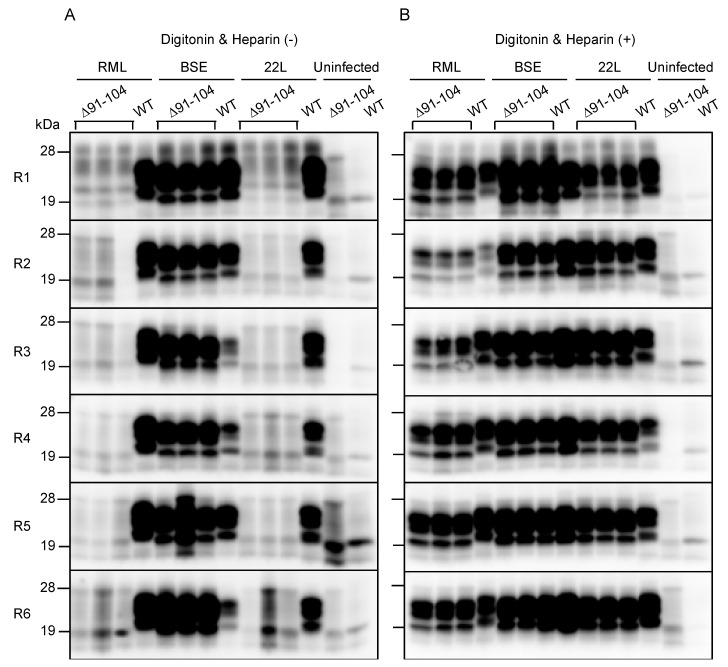
Protein misfolding cyclic amplification (PMCA) of PrP^Sc^∆91–104. Baculovirus-derived recombinant WT PrP and PrP∆91–104 were mixed with brain homogenates from RML-, BSE- and 22L-infected, terminal ill WT mice and subjected to PMCA from round 1 (R1) to R6 in the absence (**A**) or the presence (**B**) of digitonin and heparin. Uninfected brains were used as a negative control. 1/20 volume of each PMCA sample was then investigated for PrP^Sc^ and PrP^Sc^∆91–104 using western blotting (12% SDS-PAG) with T2 anti-PrP antibody after PK treatment.

The introduction of a series of mouse-chicken chimeric PrP molecules into RML-infected N2a cells or ScN2a cells, has previously suggested that residues 100–104 are crucial for RML prions to convert mouse PrP^C^ into PrP^Sc^ in cell cultures [16]. Therefore, we investigated whether PrP with a deletion from residues 91 to 104 or PrP∆91–104, could be converted into PrP^Sc^∆91–104 after infection with BSE prions but not RML prions. To this end, baculovirus-derived recombinant mouse WT PrP and PrP∆91–104 were produced and subjected to an in vitro PMCA assay with brain homogenates from RML-, BSE- and 22L-infected, terminally ill WT mice in the presence or absence of digitonin and heparin, both of which are known to enhance conversion of PrP^C^ into PrP^Sc^ in PMCA [17,18]. The resulting PMCA products in each round were investigated for PrP^Sc^ and PrP^Sc^∆91–104 after digestion with PK in western blotting with T2 anti-PrP antibody, which recognizes residues 132–156 and 212–217 of mouse PrP [19]. No PrP^Sc^ and PrP^Sc^∆91–104 were produced from WT PrP and PrP∆91–104 after incubation with control uninfected brain homogenates, even in the presence of digitonin and heparin (Figure 5A,B and Supplementary Figure S5A–D). However, the RML-, BSE- and 22L-infected brain homogenates induced the conversion of WT PrP into PrP^Sc^ even in the absence of digitonin and heparin (Figure 5A,B and Supplementary Figure S5A–D). Furthermore, PrP∆91–104 was also converted into PrP^Sc^∆91–104 after incubation with BSE-infected brain homogenates but not with RML- and 22L-infected brain homogenates, in the absence of digitonin and heparin (Figure 5A and Supplementary Figure S5A,B). However, digitonin and heparin stimulated the conversion of PrP∆91–104 into PrP^Sc^∆91–104 even after incubation with RML- and 22L-infected brain homogenates (Figure 5B and Supplementary Figure S5C,D). These results suggest that the deletion of residues 91–104 in PrP∆91–106 may be responsible for the prion strain-dependent resistance of Tg(PrP∆91–106)/*Prnp^0/0^* mice and that digitonin and heparin may play roles similar to residues 91–104 in the conversion of PrP^C^ to PrP^Sc^.

## 3. Discussion

In the present study, to determine the role of residues 91–106 of PrP^C^ in prion pathogenesis, we established a line of Tg(PrP∆91–106)/*Prnp^0/0^* mice. The mice showed a 0.4-fold expression of mouse PrP with a deletion of residues 91–106 or PrP∆91–106, in the brain compared to PrP^C^ in WT mice. PrP∆91–106 and PrP^C^ were expressed similarly at the cell surface, namely preferentially at the raft membrane microdomains, in N2a cells and in mouse brains, indicating that the deletion of residues 91–106 did not alter the subcellular localization of PrP∆91–106 at the raft domains, which are considered a major cellular site for the conversion of PrP^C^ into PrP^Sc^. However, Tg(PrP∆91–106)/*Prnp^0/0^* mice were found to be resistant to RML, 22L and FK-1 prions, neither accumulating PrP^Sc^∆91–106 or prions in the brain nor developing disease after intracerebral inoculation with RML, 22L and FK-1 prions. In contrast, they remained marginally susceptible to BSE prions, accumulating PrP^Sc^∆91–106 and prions in the brain and developing disease after long incubation times (538 ± 24 dpi). These results suggest that residues 91–106 of PrP^C^ are essential for RML, 22L and FK-1 prions but not for BSE prions, to induce the conversion of PrP^C^ into PrP^Sc^ and eventually cause prion disease in mice, although the lower expression of PrP∆91–106 in the brain might be partly responsible for the longer incubation times in BSE-infected Tg(PrP∆91–106)/*Prnp^0/0^* mice. Our results are consistent with those that PrP∆32–80 [8] and PrP∆OR [9,10], both of which contain intact residues 91–106, restored full susceptibility to RML prions in *Prnp^0/0^* mice, although *Prnp^0/0^* mice expressing PrP∆32–93 or PrP∆32–106, which lacks residues 91–106 partially or entirely, markedly reduced or completely lost their susceptibility to RML prions, respectively [11,12].

Upon conversion into PrP^Sc^, PrP^C^ undergoes marked conformational changes in the 2/3 C-terminal domain to form a PK-resistant structure [20,21]. However, the PK-cleavage site varies among PrP^Sc^ molecules derived from different prion strains. It is thus postulated that PrP^Sc^ can adopt a conformation in a prion strain-specific way and that the strain-specific pathogenic properties of prions can be enciphered in the strain-specific conformation of PrP^Sc^ [20,21]. PrP^Sc^ usually has a major PK cleavage site either in the C-terminal end of the OR region or in the post-OR region [22]. BSE-PrP^Sc^ has a major PK cleavage site between residues 95 and 96 in the post-OR region [22], resulting in a shorter PK-resistant C-terminal fragment in western blotting. By contrast, RML-, 22L and FK-1-PrP^Sc^ have a longer PK-resistant C-terminal fragment, indicating that the post-OR region is conserved in the PK-resistant core of RML-, 22L- and FK-1-PrP^Sc^. Thus, RML, 22L and FK-1 prions may favor the post-OR residues more than BSE prions for the conversion of PrP^C^ into PrP^Sc^. As a result, PrP∆91-106 is able to convert into PrP^Sc^∆91–106 in Tg(PrP∆91–106)/*Prnp^0/0^* mice inoculated with BSE prions but not with RML, 22L and FK-1 prions. Alternatively, according to the conformational selection model, inoculated PrP^Sc^ selects host PrP^C^ as a substrate for conversion on the basis of its conformational compatibility with the host PrP^C^, thereby converting the host PrP^C^ into PrP^Sc^ in a strain-dependent manner [20,21]. It is therefore also possible that, due to the deletion of residues 91–106, PrP∆91–106 may adopt a conformation that is not compatible with RML-, 22L- and FK-1-PrP^Sc^ but which is marginally compatible with BSE-PrP^Sc^. As a result, PrP∆91–106 may be slightly more convertible into PrP^Sc^∆91–106 in Tg(PrP∆91–106)/*Prnp^0/0^* mice after inoculation with BSE-PrP^Sc^ but not with RML-, 22L- and FK-1-PrP^Sc^. Moreover, we observed that secondary inoculation with PrP^Sc^∆91–106-BSE prions into Tg(PrP∆91–106)/*Prnp^0/0^* mice caused disease much earlier than Tg(PrP∆91–106)/*Prnp^0/0^* mice inoculated with WT PrP^Sc^-BSE prions (343 ± 28 vs. 538 ± 24 dpi). In contrast, WT mice inoculated with PrP^Sc^∆91–106-BSE prions developed disease much later than WT mice inoculated with WT PrP^Sc^-BSE prions (326 ± 29 vs 174 ± 4 dpi). These results suggest that PrP∆91–106 and WT PrP^Sc^ prions create so-called prion transmission barriers, which are often observed between the PrP^C^ in recipient animals and PrP^Sc^ in an inoculum when their primary sequences vary [23]. It is thus also possible that the prion transmission barrier created between PrP∆91–106 and WT BSE-PrP^Sc^ prions may be substantial but not so high, compared to those between PrP∆91–106 and WT RML-, 22L- or FK-1-PrP^Sc^-prions. This results in PrP∆91–106 being able to support BSE infection but not infection with RML, 22L and FK-1 prions. To further characterize BSE-PrP^Sc^∆91–106 prions, it would be interesting to perform the third inoculation of BSE-PrP^Sc^∆91–106 prions into Tg(PrP∆91–106)/*Prnp^0/0^* mice.

We showed that BSE-PrP^Sc^ but not RML- and 22L-PrP^Sc^, converted PrP∆91–104 into PrP^Sc^∆91–104 in PMCA, while digitonin and heparin stimulated the conversion of PrP∆91–104 into PrP^Sc^∆91–104 in PMCA even with RML- and 22L-PrP^Sc^. These results suggest that the deletion of residues 91–104 in PrP∆91–106 may be responsible for the prion strain-dependent resistance of Tg(PrP∆91–106)/*Prnp^0/0^* mice and that digitonin and heparin may play a role similar to that of residues 91–104 in the conversion of PrP^C^ to PrP^Sc^. Digitonin, a non-ionic detergent, has been shown to enhance the conversion of PrP^C^ into PrP^Sc^ in PMCA, as well as prevent PrP^C^ from forming autonomous aggregates, thereby increasing the levels of free, soluble PrP^C^ and facilitating the conversion of PrP^C^ into PrP^Sc^ in PMCA [17]. Thus, it is possible that residues 91–106 may also be involved in the structural stability of PrP^C^, preventing PrP^C^ from becoming structurally unstable enough to form autonomous aggregates. Heparin has been also reported to enhance the conversion of PrP^C^ into PrP^Sc^ in PMCA [18]. Heparin binds to residues 23–52, 53–93 and 110–128 of PrP^C^ and to the PK-resistant core of PrP^Sc^ via sulfated groups [18,24]. A previous study reported that heparin promoted the formation of amyloid fibrils from a human PrP peptide comprised of residues 106–126, indicating that heparin may function as a scaffold for the formation of amyloid fibers from peptides [25]. Since all of the heparin-binding sites of PrP∆91–104 are intact, it is possible that PrP∆91–104 is assembled on the heparin scaffold and converted into PrP^Sc^∆91–104 in PMCA and that residues 91–106 may be involved in the assembly of the PrP^C^ molecules themselves or in PrP^C^ and PrP^Sc^ upon the conversion of PrP^C^ into PrP^Sc^.

Residues 91–106 encompass the majority of the positively-charged central region, which is rich in lysine residues. This region was previously demonstrated to be involved in the lipid-induced conversion of recombinant PrP into PK-resistant PrP [26]. It is thus possible that the positive charge in residues 91–106 may be important for PrP^C^ molecules either to be structurally stable and soluble or to assemble with themselves and/or with PrP^Sc^. Alternatively, residues 91–106 contain proline residues, which are mutated to leucine residues in human hereditary prion diseases (P101L and P104L in mouse PrP homologous to P102L and P105L in human PrP). These mutations are considered to trigger structural changes in the surrounding region, eventually causing the structural instability of the mutant PrPs and thereby triggering the conversion of mutant PrPs into pathogenic PrPs [27]. Therefore, it is possible that residues 91–106 play an important role in the structural integrity of PrP^C^. Furthermore, the substitution of copper-binding histidine (H) residue to glycine (G) residue at position 95 in mouse PrP was reported to accelerate prion disease in mice after infection with RML prions [28], suggesting that copper-binding at H95 might also structurally stabilize PrP^C^ and thereby play an inhibitory role in the conversion of PrP^C^ into PrP^Sc^.

In summary, we showed that Tg(PrP∆91–106)/*Prnp^0/0^* mice were resistant to RML, 22L and FK-1 prions and remained marginally susceptible to BSE prions, suggesting that residues 91–106 of PrP^C^ are crucially involved in prion pathogenesis in a strain-dependent manner. We also showed that these residues might have a similar role to that of digitonin and heparin in the conversion of PrP^C^ into PrP^Sc^. The precise role of residues 91–106 in the conversion of PrP^C^ into PrP^Sc^ would be helpful not just to improve our understanding of the mechanism underlying the conversion of PrP^C^ into PrP^Sc^ but also for the development of therapeutic agents in prion diseases.

## 4. Materials and Methods

### 4.1. Ethics Statements

The Ethics Committees of Animal Care and Experimentation of the University of Occupational and Environmental Health (approval number AE08-013, 18 March 2019), Kyoto University (approval number S-13-10-2, 1 April 2017) and the Institutional Animal Care and Use Committee at the National Institute of Animal Health (approval number 13-005, 13 April 2013) approved this study. Animals were cared for in accordance with The Guiding Principle for Animal Care and Experimentation of the University of Occupational and Environmental Health, Kyoto University, the National Institute of Animal Health and Japanese Law for Animal Welfare and Care. Every effort was made to reduce distress and the number of animals used.

### 4.2. Generation of Tg(PrP∆91–106)/Prnp^0/0^ Mice

A DNA fragment encoding residues 1–90 fused with residues 107–111 of mouse PrP was amplified by polymerase chain reaction (PCR) with a sense primer (5′-cccgtcgacctcgagatggcgaaccttggc-3′; underlined sequence, *Sal* I and *Xho* I sites; bold sequence, start codon) and an antisense primer (5′-cacatgcttgaggtt*ttggccccatccacc*-3′; underlined sequence, residues 107–111; italic sequence, residues 86–90) using mouse WT PrP^C^-encoding pcDNA3.1-moPrP [29] as a template. The resulting DNA fragment was then utilized as a 5′ primer to amplify another DNA fragment encoding PrP∆91–106 together with an antisense primer (5′-cccgtcgacctcgagtcatcccacgatcag-3′; underlined sequence, *Sal* I and *Xho* I sites; bold sequence, stop codon) using pcDNA3.1-moPrP as a template. The amplified DNA was then cloned into pCR2.1-TOPO TA vector (Thermo Fisher Scientific, Waltham, MA, USA), resulting in pCR2.1-TOPO TA-PrP∆91–106. After DNA sequence confirmation, it was inserted into a unique *Sal* I site of the Syrian hamster PrP cosmid vector, CosSHa.tet (InPro Biotechnology, Inc., South San Francisco, CA, USA), to construct PrP∆91–106 transgene. The transgene was injected into the zygotes of C57BL/6 mice, as described elsewhere [30,31], after the removal of the cosmid-derived sequences, resulting in a line of Tg(PrP∆91–106) mice. The Tg(PrP∆91–106) mice were successively mated with *Prnp^0/0^* mice, which had been intercrossed with C57BL/6 mice more than 10 times [32,33], to produce Tg(PrP∆91–106)/*Prnp^0/0^* mice.

### 4.3. Prion Inoculation

Brains from terminally ill C57BL/6 mice infected with RML, 22L, FK-1 and BSE prions were homogenized (10%, *w*/*v*) in phosphate-buffered saline (PBS) by passing through 18 to 26 gauge needles and then diluted to 1% with PBS. Four to five week-old Tg(PrP∆91–106)/*Prnp^0/0^* and C57BL/6 mice (CLEA Japan, Tokyo, Japan) were intracerebrally inoculated with a 20 μL-aliquot of the homogenates.

### 4.4. Establishment of N2a∆PrP/PrP∆91–106 Cells

The *BamH* I/*Xba* I DNA fragment including mouse PrP∆91–106 from pCR2.1-TOPO TA-PrP∆91–106 was inserted into pcDNA3.1(+) (Thermo Fisher Scientific) to construct an expression vector for PrP∆91–106, designated pcDNA3.1(+)-PrP∆91–106. The vector was transfected into PrP^C^-knockout N2a∆PrP cells [14] using Lipofectamine 2000 (Thermo Fisher Scientific). The transfected cells were then subjected to neomycin selection followed by a limiting dilution to produce N2a∆PrP/PrP∆91–106 cells.

### 4.5. Western Blotting

Tissues were homogenized (10%, *w*/*v*) using a Multi-beads shocker (Yasui Kikai Co., Osaka, Japan) in lysis buffer (150 mM NaCl, 50 mM Tris-HCl, pH 7.5, 0.5% Triton X-100, 0.5% sodium deoxycholate, 1 mM EDTA) containing protease inhibitor mixture (Nakalai Tesque Co., Kyoto, Japan) and protein concentrations were determined using the BCA protein assay kit (Pierce, Rockford, IL, USA). The homogenates were then subjected to an SDS-polyacrylamide gel electrophoresis after treatment with or without PK (Wako Pure Chemical Industries, Ltd., Osaka, Japan) at 20 µg/mL for 30 min at 37 °C and then the total proteins were electrically transferred to an Immobilon-P PVDF membrane (Millipore Corp., MA, USA). The membrane was blocked using TBST (0.1% Tween-20, 100 mM NaCl, 10 mM Tris-HCl, pH7.6) containing 5% non-fat dry milk for 1 h at room temperature (RT), incubated with rabbit polyclonal anti-PrP antibody (18635; Immuno Biological Laboratories, Gunma, Japan), SAF61 mouse monoclonal antibody (Bertin Pharma, Montigny le Bretonneux, France), rabbit polyclonal anti-Flottilin-2 antibody (Cell Signaling, MA, USA) or anti-β-actin monoclonal antibody (Sigma-Aldrich, St. Louis, MO, USA) for 2 h at RT or overnight at 4 °C in TBST containing 1% non-fat dry milk and washed in TBST several times. Chemiluminescent signals were detected using horseradish peroxidase (HRP)-conjugated anti-mouse IgG antibodies (Amersham Biosciences, Piscataway, NJ, USA), anti-goat IgG antibodies (CHEMICON International., Temecula, CA, USA) and Immobilon Western Chemiluminescent HRP substrate (Millipore), with a chemiluminescence image analyzer (LAS-4000 mini; Fujifilm Co., Tokyo, Japan).

### 4.6. HE Staining

Samples embedded in paraffin were sliced at 5 μm and the sliced samples were then deparaffinized, rehydrated, stained with Mayer’s hematoxylin solution (Wako Pure Chemical Industries, Osaka, Japan) and 1% Eosin Y solution (Wako Pure Chemical Industries) and mounted with Softmount (Wako Pure Chemical Industries).

### 4.7. Immunohistochemistry

Paraffin-embedded samples were sectioned, deparaffinized and rehydrated. The samples were autoclaved in 1 mM HCl at 105 °C for 5 min and subsequently washed with PBS. For the detection of PrP^Sc^, the samples were digested with 50 μg/mL PK in PBS at 37 °C for 30 min, treated with 3 M guanidine thiocyanate for 10 min and washed with PBS. After blocking with 5% FBS in PBS for 1 h, the samples were incubated with SAF83 (Bertin Pharma, Montigny le Bretonneux, France) anti-PrP Ab or rabbit polyclonal anti-GFAP antibody (Shima laboratories Co., Ltd., Tokyo, Japan) for 2 h, washed with PBS and treated with ImmPRESS REAGENT Anti-Mouse IgG (Vector Laboratories, Burlingame, CA, USA) for 30 min. After washing with PBS, the samples were incubated with ImmPACT DAB (Vector Laboratories) for 180 s for staining of PrP^Sc^ or 60 s for staining of GFAP.

Cells grown on coverslips were fixed with 3% PFA for 10 min at room temperature and permeabilized with 0.1% Triton X-100 in PBS for 4 min at room temperature. After blocking with 5% FBS in PBS for 1 h, the cells were stained with SAF83 anti-PrP Ab (Bertin pharma) for 2 h, washed with PBS and stained with Alexa Fluoro 488-conjugated anti-mouse IgG Abs (Thermo Fisher Scientific) and 1 μg/mL DAPI (Sigma-Aldrich, St Louis, MO, USA) for 2 h at room temperature.

### 4.8. Fractionation of Membrane Microdomains

Cells grown to ~80% confluency in a 60-mm tissue culture dish were suspended in 250 μL of MBS buffer (25 mM MES-NaOH (pH 6.5), 0.15 M NaCl) containing 1% (*w*/*v*) Triton X-100 and homogenized by being passed through a 21G-needle 15 times. After centrifugation at 500× *g* for 5 min at 4 °C, 220 μL of the supernatant was transferred to a new tube and mixed with 220 μL of MBS buffer containing 80% (*w*/*v*) sucrose to make 40% (*w*/*v*) sucrose. Then, 200 μL of sample was placed at the bottom of a discontinuous sucrose gradient consisting of 600 μL of 30% (*w*/*v*) sucrose and 200 μL of 5% (*w*/*v*) sucrose and centrifuged at 140,000× *g* for 24 h at 4 °C in an S55S rotor (Hitachi Koki, Tokyo, Japan). Ten fractions (100 μL/fraction) were collected from the top.

### 4.9. Preparation of Insect Cell Suspension Expressing Recombinant WT PrP and PrP∆91–104

A baculovirus expression vector for mouse WT PrP, termed pFastBac-moPrP, was used as constructed elsewhere [34] and a baculovirus expression vector for PrP∆91–104, pFastBac-moPrP∆91–104, was constructed in the present study. In brief, a DNA fragment encoding residues 1–90 fused with residues 105–109 of mouse PrP was amplified by PCR with a Met forward sense primer (5′-cgggatccgccacc**atg**gcgaaccttggctac-3′; underlined sequence, *BamH* I site; bold sequence, start codon) and a moPrP∆91–104 antisense primer (5′-gaggttggtttt*ttggccccat*-3′; underlined sequence, residues 105–108; italic sequence, residues 88–90) using pFastBac-moPrP as a template. The resulting DNA fragment was then utilized as a 5′ primer to amplify another DNA fragment encoding PrP∆91–104 together with a Stop reverse antisense primer (5′-cccaagctt**tca**tcccacgatcaggaag-3′; underlined sequence, *Hind* III site; bold sequence, stop codon) using pFastBac-moPrP as a template. The amplified DNA were cloned into pCR2.1-TOPO TA vector (Thermo Fisher Scientific). After confirming the DNA sequence of the fragments, these were inserted into the *BamH* I/*Hind* III-digested baculovirus expression vector pFastBac1 (Thermo Fisher Scientific).

Recombinant WT PrP and PrP∆91–104 were prepared according to the manufacturer’s protocol (Thermo Fisher Scientific). pFastBac-moPrP(3F4) and pFastBac-moPrP∆91–104 were first introduced into DH10 Bac *E. coli* to obtain recombinant bacmid encoding each molecule. The bacmid was then transfected into *Spodoptera frugiperda* 21 insect cells with a bacmid DNA-cellfectin mixture (Thermo Fisher Scientific) to obtain recombinant baculovirus encoding WT PrP or PrP∆91–104. HighFive cells (Thermo Fisher Scientific) were infected with the recombinant baculovirus encoding each molecule for 72 h at 27 °C to produce recombinant WT PrP or PrP∆91–104. The cells were suspended in distilled water at a concentration of 5 × 10^4^ cells/µL for PMCA. The yield of recombinant WT PrP or PrP∆91–104 in the cells was approximately 16 µg/10^6^ cells.

### 4.10. PMCA

The brains from RML-, 22L- or BSE-infected, terminal ill ICR mice were homogenized at a 20% (*w*/*v*) concentration in PBS containing a complete protease inhibitor cocktail (Roche) and then diluted to 10% by adding an equal volume of 2× PMCA buffer (2× PBS, 8 mM EDTA, 2% Triton X-100). Uninfected ICR mouse brains were prepared in a similar manner as a control. ICR mice were purchased from Japan SLC Inc. (Hamamatsu, Japan). Briefly, 5 µL of cell suspension (5 × 10^4^ cells/µL) producing WT PrP or PrPΔ91–104 was added to 95 µL of each of the 10% brain homogenates and subjected to PMCA procedures. PMCA was performed using automatic cross-ultrasonic protein activating apparatus (Elestein 070-GOT; Elekon Science Corp., Chiba, Japan), as previously reported [34]. Amplification was performed using 32 cycles of sonication (pulse oscillation for 3 s repeated five times at 0.1-s intervals), followed by incubation at 37 °C for 30 min with gentle agitation. The amplified products of the first round of amplification were diluted 1:10 with the WT PrP or PrP∆91–104 cell suspension and subjected to a second round of amplification. This process was repeated up to 6 rounds. The products from each round were treated with 40 µg/mL of PK for 1 h at 37 °C and subjected to western blotting with HRP-conjugated T2 anti-PrP antibody [19]. Signals were detected using SuperSignal West Dura Extended Duration Substrate (Pierce) with a ChemiImager (Alpha InnoTec, San Leandro, CA, USA).

## Figures and Tables

**Table 1:** Incubation times in WT and Tg(PrP∆91–106)/*Prnp^0/0^* mice inoculated with RML, 22L, FK-1 and bovine spongiform encephalopathy (BSE) prions.

Prions	Recipient Mouse	Expression Level of PrP (Fold) ^1^	Diseased Mice/Total Mice	Times to the Onset of Disease (Days)
RML	WT	1	8/8	171 ± 4
	Tg(PrP∆91–106)/*Prnp^0/0^*	0.4	0/11	>765
22L	WT	1	10/10	143 ± 1
	Tg(PrP∆91–106)/*Prnp^0/0^*	0.4	0/9	>623
FK-1	WT	1	10/10	187 ± 3
	Tg(PrP∆91–106)/*Prnp^0/0^*	0.4	0/4	>604
BSE	WT	1	10/10	174 ± 4
	Tg(PrP∆91–106)/*Prnp^0/0^*	0.4	10/10	538 ± 24

^1^ Expression levels of mutant PrP were compared to those of PrP^C^ in WT mice using Western blotting.

**Table 2:** Secondary transmission of PrP^Sc^∆91–106 prions in WT and Tg(PrP∆91–106)/*Prnp^0/0^* mice.

Prions	Donor Mouse	Recipient Mouse	Diseased Mice /Total Mice	Incubation Times ^1^ (Days)
RML	Tg(PrP∆91–106)/*Prnp^0/0^*	WT	0/6	>730
		Tg(PrP∆91–106)/*Prnp^0/0^*	0/4	>730
22L	Tg(PrP∆91–106)/*Prnp^0/0^*	WT	0/9	>732
		Tg(PrP∆91–106)/*Prnp^0/0^*	0/4	>732
FK-1	Tg(PrP∆91–106)/*Prnp^0/0^*	WT	0/6	>730
		Tg(PrP∆91–106)/*Prnp^0/0^*	0/6	>730
BSE	Tg(PrP∆91–106)/*Prnp^0/0^*	WT	10/10	326 ± 29
		Tg(PrP∆91–106)/*Prnp^0/0^*	9/9	343 ± 28

^1^ Times to the onset of disease.

## Supplementary Materials

Supplementary materials can be found at https://www.mdpi.com/1422-0067/21/19/7260/s1, Figure S1: Original, uncropped and unadjusted images of Figure 1B, Figure S2: (A) Original, uncropped and unadjusted images of Figure 2A. (B) Original, uncropped and unadjusted images of Figure 2C. (C) Original, uncropped and unadjusted images of Figure 2D, Figure S3: (A–D) Original, uncropped and unadjusted images of Figure 3A–D, Figure S4: (A) HE staining and immunohistochemistry for WT PrP^Sc^ and PrP^Sc^∆91–106 with SAF83 anti-PrP antibody of the brains of RML-infected, terminally ill WT mice and Tg(PrP∆91–106)/*Prnp^0/0^* mice sacrificed at 573 dpi with RML prions. (B) HE staining and immunohistochemistry for WT PrP^Sc^ and PrP^Sc^∆91–106 with SAF83 anti-PrP antibody of the brains of 22L-infected, terminally ill WT mice and Tg(PrP∆91–106)/*Prnp^0/0^* mice sacrificed at 603 dpi with 22L prions. (C) HE staining and immunohistochemistry for WT PrP^Sc^ and PrP^Sc^∆91–106 with SAF83 anti-PrP antibody of the brains of FK-1-infected, terminally ill WT mice and Tg(PrP∆91–106)/*Prnp^0/0^* mice sacrificed at 603 dpi with FK-1 prions, Figure S5: (A,B) Original, uncropped and unadjusted images of Figure 5A. (C,D) Original, uncropped and unadjusted images of Figure 5B.
