# Supplementary material for: Strain-Dependent Prion Infection in Mice Expressing Prion Protein with Deletion of Central Residues 91–106
Source: Int J Mol Sci. 2020 Oct 1;21(19):7260. doi: 10.3390/ijms21197260 (PMC7582732; doi:10.3390/ijms21197260)
Supplement: Supplementary file 1 [file ijms-21-07260-s001.pdf]

Supplementary Materials

Strain-Dependent Prion Infection in Mice Expressing Prion Protein with Deletion of Central Residues 91–106

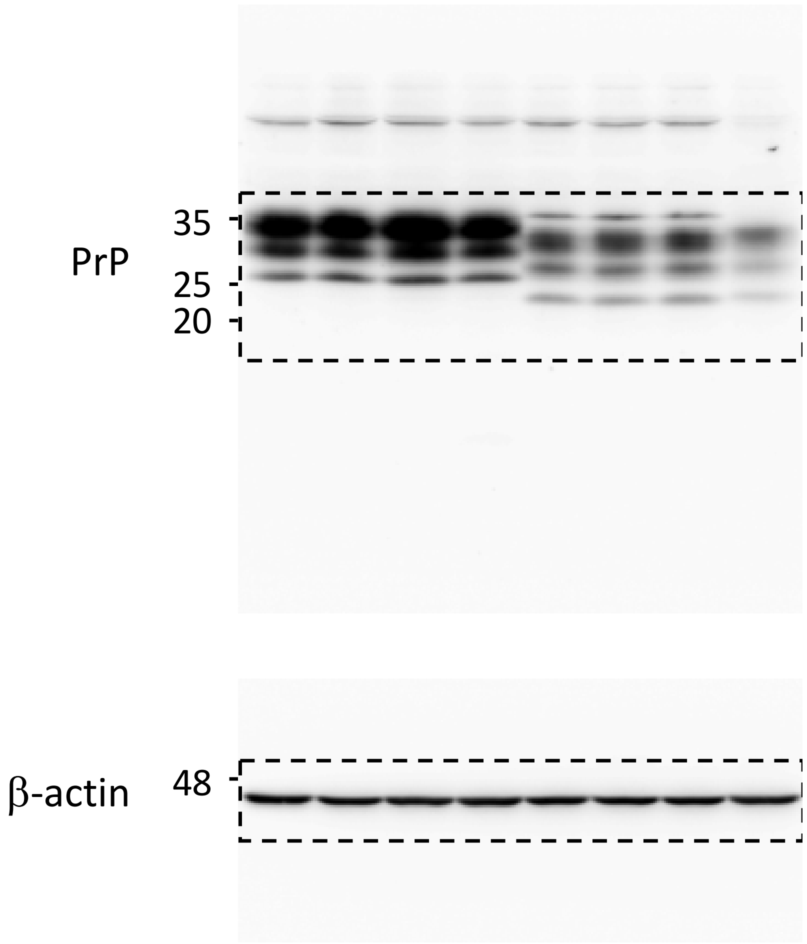

Figure S1. Original, uncropped and unadjusted images of Figure 1B.

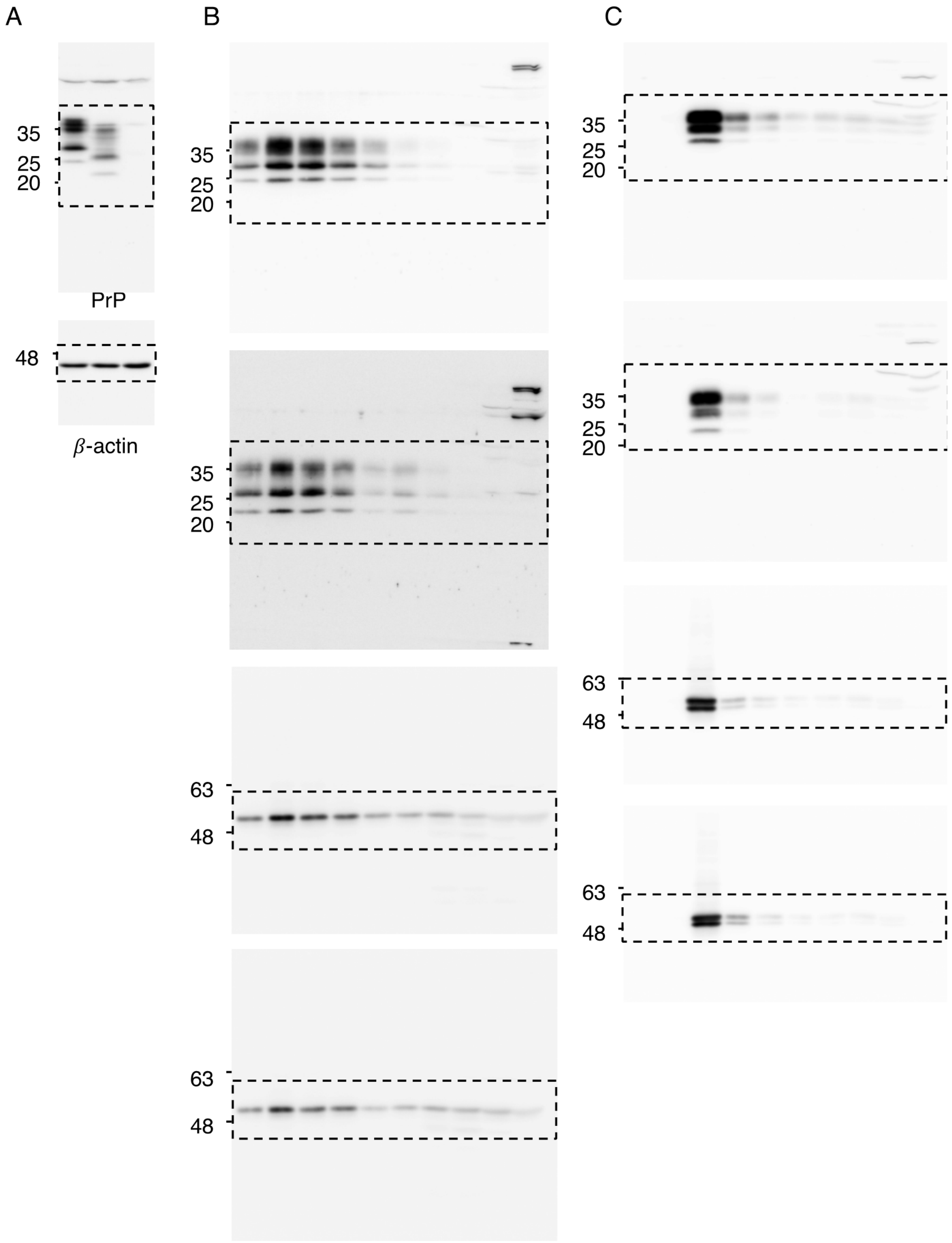

Figure S2. (**A**) Original, uncropped and unadjusted images of Figure 2A. (**B**) Original, uncropped and unadjusted images of Figure 2C. (**C**) Original, uncropped and unadjusted images of Figure 2D.

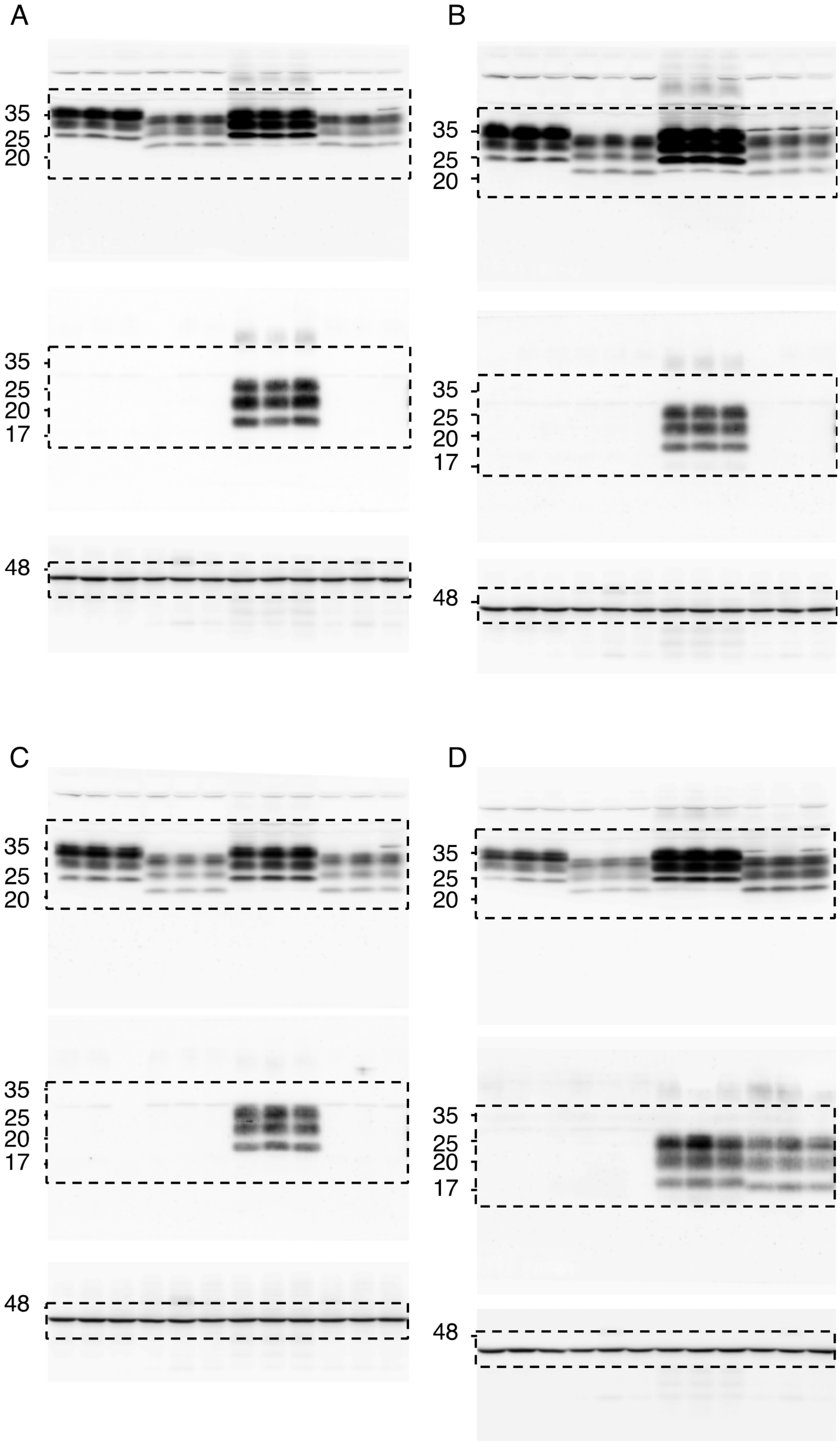

Figure S3. (**A**–**D**) Original, uncropped and unadjusted images of Figure 3A–D.

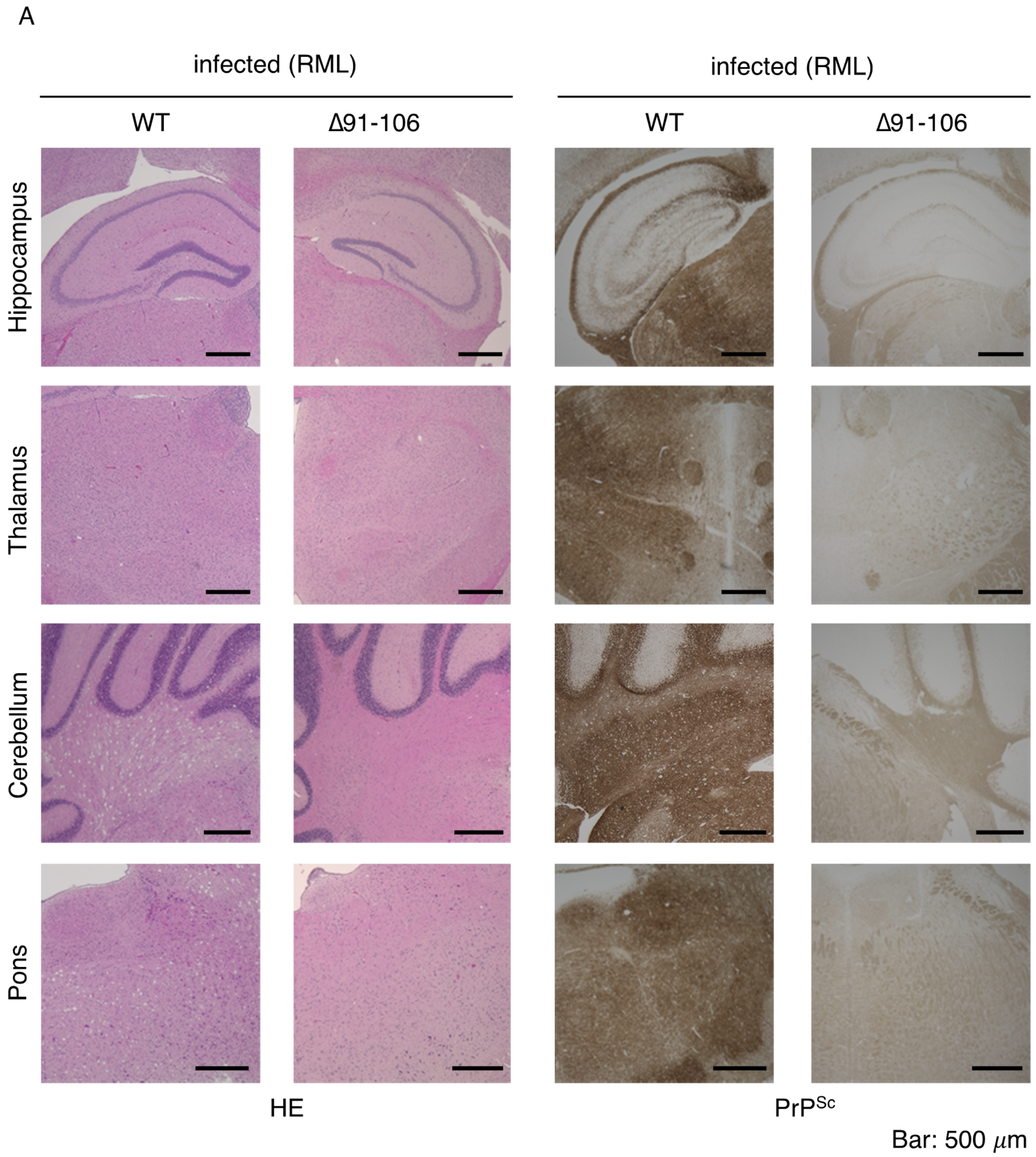

Figure S4. *Cont.*

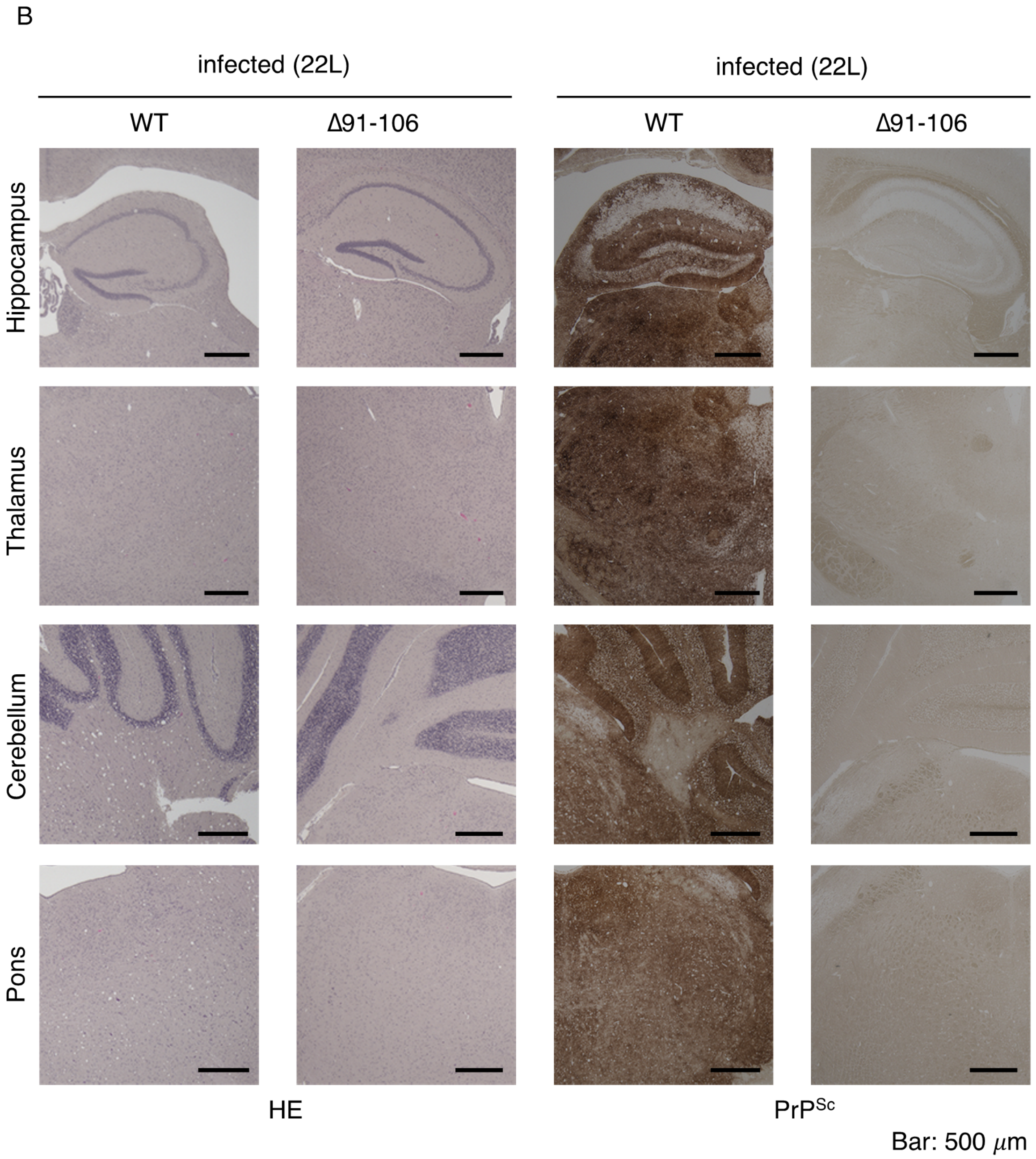

Figure S4. *Cont.*

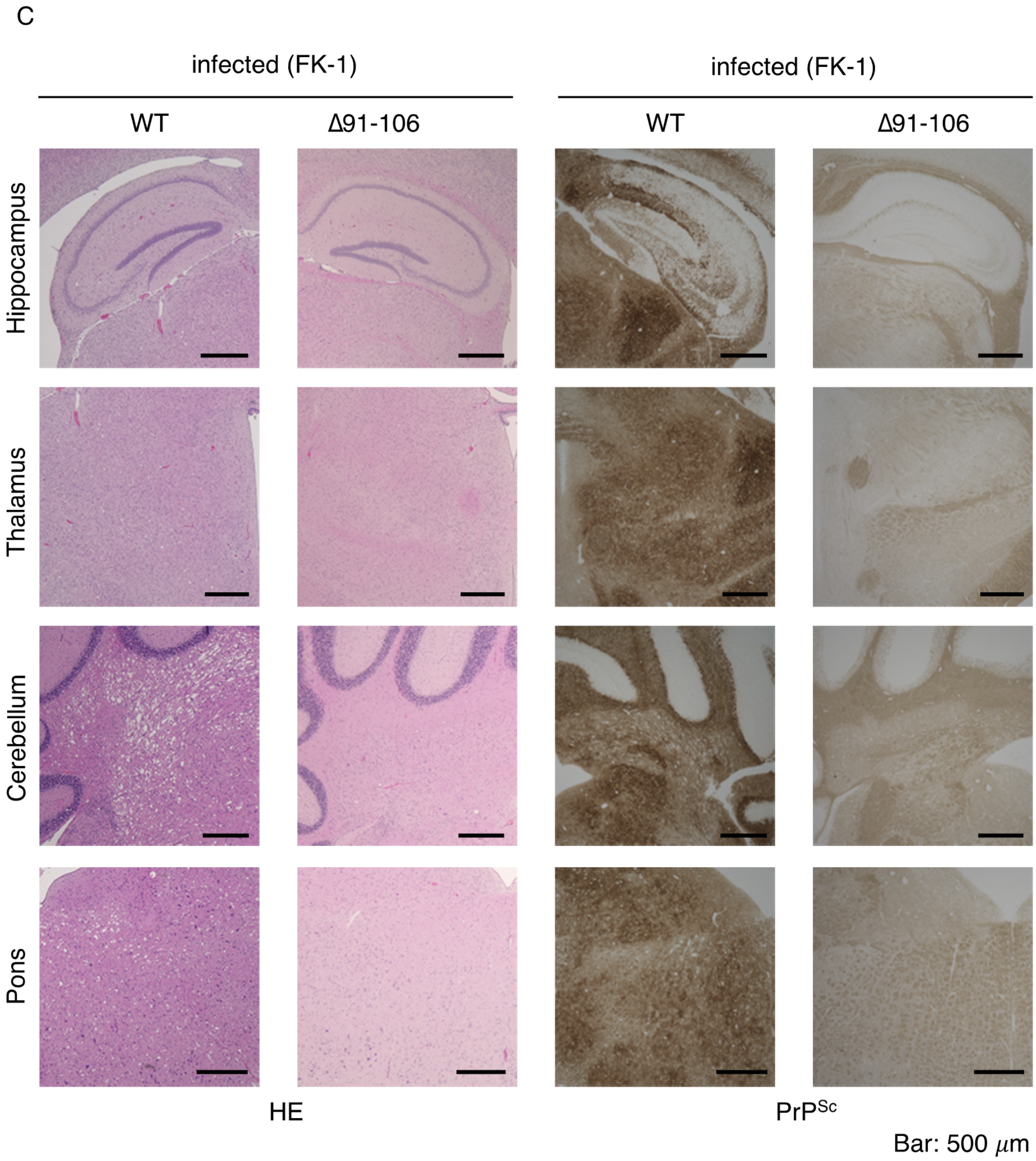

Figure S4. (**A**) HE staining and immunohistochemistry for WT PrP^{Sc} and $PrP^{Sc}\Delta 91–106$ with SAF83 anti-PrP antibody of the brains of RML-infected, terminally ill WT mice and Tg(PrPΔ91–106)/$Prnp^{0/0}$ mice sacrificed at 573 dpi with RML prions. (**B**) HE staining and immunohistochemistry for WT PrP^{Sc} and $PrP^{Sc}\Delta 91–106$ with SAF83 anti-PrP antibody of the brains of 22L-infected, terminally ill WT mice and Tg(PrPΔ91–106)/$Prnp^{0/0}$ mice sacrificed at 603 dpi with 22L prions. (**C**) HE staining and immunohistochemistry for WT PrP^{Sc} and $PrP^{Sc}\Delta 91–106$ with SAF83 anti-PrP antibody of the brains of FK-1-infected, terminally ill WT mice and Tg(PrPΔ91–106)/$Prnp^{0/0}$ mice sacrificed at 603 dpi with FK-1 prions.

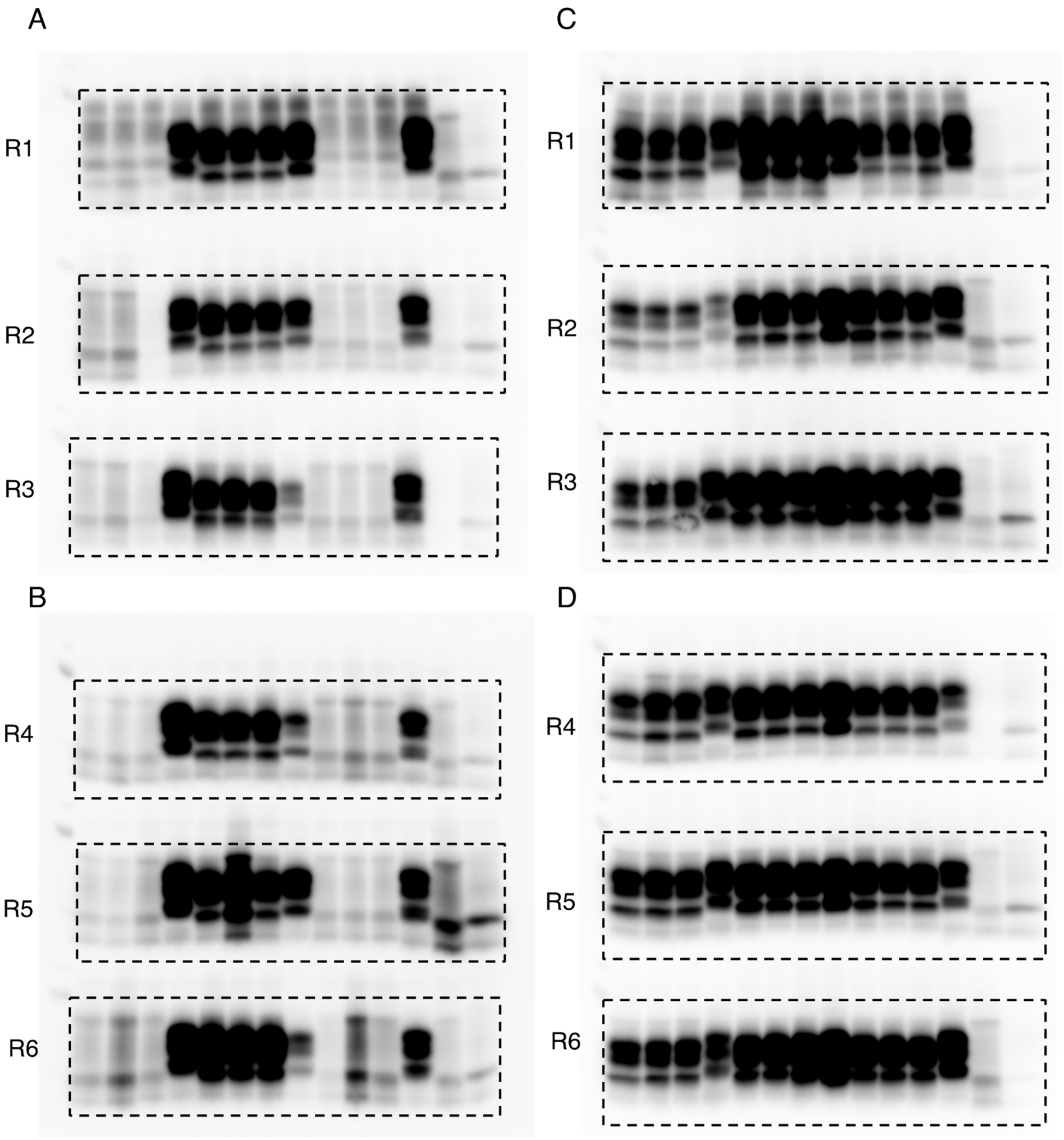

Figure S5. (**A**,**B**) Original, uncropped and unadjusted images of Figure 5A. (**C**,**D**) Original, uncropped and unadjusted images of Figure 5B.
